# The Cancer Omics Atlas: an integrative resource for cancer omics annotations

**DOI:** 10.1186/s12920-018-0381-7

**Published:** 2018-08-08

**Authors:** Qingrong Sun, Mengyuan Li, Xiaosheng Wang

**Affiliations:** 1 Department of Basic Medicine, School of Basic Medicine and Clinical Pharmacy, China Pharmaceutical University, Nanjing, 211198 China; 2 Biomedical Informatics Research Lab, School of Basic Medicine and Clinical Pharmacy, China Pharmaceutical University, Nanjing, 211198 China; 3 Cancer Genomics Research Center, School of Basic Medicine and Clinical Pharmacy, China Pharmaceutical University, Nanjing, 211198 China; 4 Big Data Research Institute, China Pharmaceutical University, Nanjing, 211198 China

**Keywords:** Cancer genomics, microRNA, Proteomics, Pan-cancer, Immuno-oncology

## Abstract

**Background:**

The Cancer Genome Atlas (TCGA) is an important data resource for cancer biologists and oncologists. However, a lack of bioinformatics expertise often hinders experimental cancer biologists and oncologists from exploring the TCGA resource. Although a number of tools have been developed for facilitating cancer researchers to utilize the TCGA data, these existing tools cannot fully satisfy the large community of experimental cancer biologists and oncologists without bioinformatics expertise.

**Methods:**

We developed a new web-based tool The Cancer Omics Atlas (TCOA, http://tcoa.cpu.edu.cn) for fast and straightforward querying of TCGA “omics” data.

**Results:**

TCOA provides the querying of gene expression, somatic mutations, microRNA (miRNA) expression, protein expression data based on a single molecule or cancer type. TCOA also provides the querying of expression correlation between gene pairs, miRNA pairs, gene and miRNA, and gene and protein. Moreover, TCOA provides the querying of the associations between gene, miRNA, or protein expression and survival prognosis in cancers. In addition, TCOA displays transcriptional profiles across various human cancer types based on the pan-cancer analysis. Finally, TCOA provides the querying of molecular profiles for 2877 immune-related genes in human cancers. These immune-related genes include those that are established or promising targets for cancer immunotherapy such as *CTLA4*, *PD1*, *PD-L1*, *PD-L2*, *IDO1*, *LAG3,* and *TIGIT*.

**Conclusions:**

TCOA is a useful tool that supplies a number of unique and new functions complementary to the existing tools to facilitate exploration of the TCGA resource.

## Background

With the development of high-throughput sequencing technology, a large volume of cancer genomics data are emerging and advancing cancer research. Notably, The Cancer Genome Atlas (TCGA) datasets cover 33 different cancer types and more than 10,000 cancer cases in total (https://gdc-portal.nci.nih.gov/). Each TCGA cancer type contains different types of “omics” data, including: whole exome (genome) sequencing; genomic DNA copy number arrays; DNA methylation; mRNA expression array and RNA-Seq data; microRNA (miRNA) sequencing; reverse-phase protein arrays; and clinical metadata. TCGA is becoming a necessary data resource not only for the cancer informatics researchers, but also for experimental cancer researchers and oncologists. Particularly, many cancer researchers are interested in having a preliminary search of TCGA to find or filter their experimental targets; many researchers seek for a validation of their experimental results from TCGA. However, because most of experimental biologists and oncologists lack sufficient skills in bioinformatics analysis, it is usually difficult for them to explore the TCGA resource. Thus, the development of web-based tools with the user-friendly graphical user interface (GUI) must be useful for experimental biologists and oncologists to search what they need from TCGA.

Some web-based tools have been developed to explore the TCGA data. The cBioPortal (http://cbioportal.org) is a web resource for analyzing and visualizing multidimensional cancer genomics data including those from TCGA [1, 2]. The Broad Institute TCGA GDAC Firehose (http://gdac.broadinstitute.org/) provides standardized datasets, algorithms, and analysis results for TCGA. MEXPRESS (http://mexpress.be/) provides query and visualization of the clinical, gene expression and methylation data in TCGA [3]. GEPIA (http://gepia.cancer-pku.cn/) is a web tool for visualizing gene expression comparisons and correlations, and associations with patient survival prognosis based on TCGA and GTEx data [4]. The Cancer Proteome Atlas (http://tcpaportal.org/tcpa/) is a web-based data portal for downloading, visualizing, and analyzing TCGA proteomics data [5]. All these tools provide significantly valuable resources that facilitate cancer biologists and oncologists to explore the TCGA data. However, the existing tools still have many places worth improving to satisfy the large community of experimental biologists and oncologists without bioinformatics expertise. For example, cBioPortal lacks differential expression and survival analyses based on gene expression profiles while these data are often of interests for biologists and oncologists. GDAC Firehose is a good resource for bioinformatics scientist while is not straightforward for cancer biologists and oncologists without bioinformatics training. Although MEXPRESS can provide the fast querying of the visualized clinical, gene expression and methylation data in TCGA, it lacks some important data types that are relevant to cancer biology and oncology such as gene somatic mutations, miRNAs, proteins and their associations with survival prognosis in cancers. Similarly, GEPIA is a recently-published web tool that specializes in querying gene expression and its association with survival prognosis in cancers while it lacks other cancer omics data such as gene somatic mutations, miRNAs and proteomics, and the associations of these molecular profiles with survival prognosis in cancers.

To provide useful functions complementary to these existing tools, we developed a new web-based tool The Cancer Omics Atlas (TCOA, http://tcoa.cpu.edu.cn) for fast and straightforward querying of TCGA gene expression, somatic mutations, miRNA expression, protein expression based on a single molecule or cancer type. TCOA also provides the querying of expression correlations of gene-gene, miRNA-miRNA, protein-protein, gene-miRNA and gene-protein, and the correlation of gene, miRNA, or protein expression with survival prognosis in cancers. Moreover, TCOA provides a portrait of transcriptional landscape of human cancers based on the pan-cancer analysis. In addition, because cancer immunotherapy is showing increasingly noteworthy for its effectiveness in treating a variety of cancers, we specifically provide a tab for querying of 2877 immune-related genes in TCOA.

## Construction and content

### Database architecture and web interface

TCOA was developed using Hypertext Preprocessor (PHP, version 5.5.10) with a R-based web framework. The back-end database was built by MySQL (version 5.5.36) that contained the TCGA data needed for querying. PHP scripts were used to handle database queries or computational results by R script, generate results and send them to users. The TCOA website was developed by HTML (Hyper TextMarkup Language) and JavaScript for the user interface. TCOA contains six major modules: Gene, MicroRNA, Cancer, Pan-cancer, Immuno-Oncology, and Protein (Table 1). For all the querying from users, TCOA will send visualized results to them in the form of figures (a few in the form of tables).

**Table 1:** A summary of TCOA functions and data display

Module	Function	Visualization
Gene	show mean gene expression values in different cancer types	bar chart
	compare gene expression between cancer and normal samples	box plot
	show expression correlation between gene and gene in cancers	scatter diagram
	compare gene expression between different cancer phenotypes (T, N, M, Stage and Grade)	box plot
	compare survival time between gene higher-expression-level and lower-expression-level cancers	survival curve
	show gene somatic mutation rates in cancers	bar chart
	compare gene somatic mutation rates between different cancer phenotypes (T, N, M, Stage and Grade)	box plot
	classify gene somatic mutations in cancers	pie chart
	compare survival time between gene-mutated and gene-wildtype cancers	survival curve
	compare gene expression between gene-mutated and gene-wildtype cancers	box plot
MicroRNA	show mean miRNA expression values in different cancer types	bar chart
	compare miRNA expression between cancer and normal samples	box plot
	show expression correlation between gene and miRNA in cancers	scatter diagram
	show expression correlation between miRNA and miRNA in cancers	scatter diagram
	compare miRNA expression between different phenotypes (T, N, M, Stage and Grade) in cancers	box plot
	compare survival time between miRNA higher-expression-level and lower-expression-level cancers	survival curve
Cancer	show mutation rates of the 50 most frequently mutated genes in the cancer type	bar chart
	show the up-regulated and down-regulated genes in the cancer type satisfying the threshold given by users	bar chart
	show important pathways associated with the highly-expressed genes in the cancer type	bar chart
	show the up-regulated and down-regulated miRNAs in the cancer type satisfying the threshold given by users	bar chart
Pan-cancer	show pathways significantly up-regulated in cancers	bar chart
	show genes whose upregulation is associated with poor prognosis in cancers	survival curve
	show genes whose downregulation is associated with poor prognosis in cancers	survival curve
	show genes with increased or decreased expression alterations consistently from normal tissue to low-advanced cancers, and from low-advanced cancers to highly-advanced cancers	bar chart
	show the cell cycle pathway consistently up-regulated in cancers	bar chart
	show genes whose expression levels are significantly higher or lower in cancers than in normal tissue	table
	show genes whose expression levels are significantly higher or lower in high-grade cancers than in low-grade cancers	table
	show genes whose expression levels are significantly higher or lower in late-stage cancers than in early-stage cancers	table
	compare tumor mutation burden among different cancer types	bar chart
Immuno-Oncology	query molecular profiles of 2877 immune-related genes in cancers	the same as the “Gene” module
Protein	show mean protein expression levels (normalized) in different cancer types	bar chart
	show expression correlation between gene and protein in cancers	scatter diagram
	compare protein expression between different cancer phenotypes (T, N, M, Stage and Grade)	bar chart
	compare survival time between protein higher-expression-level and lower-expression-level cancers	survival curve

T: describes the size of the original (primary) tumor and whether it has invaded nearby tissue

N: describes nearby (regional) lymph nodes that are involved

M: describes distant metastasis (spread of cancer from one part of the body to another)

Stage: describes the progression of cancer

Grade: describes the differentiated level of cancer

low-advanced cancers: early-stage (Stage I-II) or low-grade (Grade I-II) cancers

highly-advanced cancers: late-stage (Stage III-IV) or high-grade (Grade III-IV) cancers

tumor mutation burden: the total number of substitutions, regardless of somatic mutation type in tumor

**Table 2:** Database statistics

Data type	Total number
cancer types	33
cancer samples	9914
normal samples	712
genes (expression)	20,531
genes (mutations)	32,774
immune genes	2877
miRNAs	1046
proteins	295

### Functions of six modules in the database

#### Functions of the “gene” module

In the “Gene” module, when a user submits the querying of a gene using the gene symbol or Entrez ID, TCOA will output the information on expression and somatic mutations of the gene in 33 cancer types. The gene expression data include: gene expression levels in cancers; expression correlations with other genes in cancers; differential expression comparisons between cancer and normal samples (if the gene expression data in normal samples are available in TCGA); differential expression comparisons between different cancer phenotypes such as stage and grade; associations of gene expression with survival prognosis in cancers. The gene somatic mutation data include: mutation rates in cancers; variants classification in cancers; comparisons of mutation rates between different cancer phenotypes such as stage and grade; comparisons of gene expression between gene-mutated and gene-wildtype cancers; associations of gene mutations with survival prognosis in cancers.

**Fig. 1:**
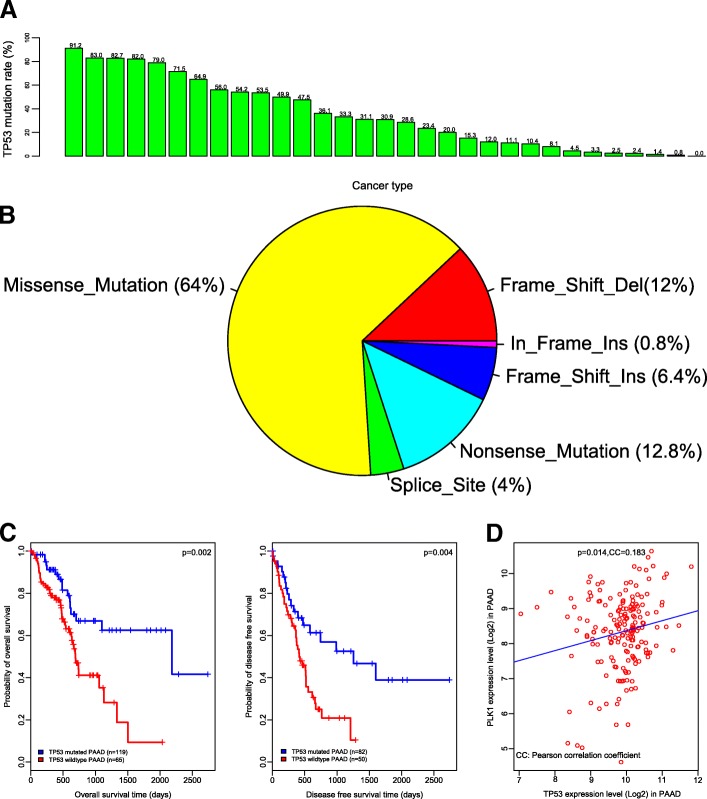
Investigation of *TP53* in the “Gene” module. **a** *TP53* mutation rates in different cancer types. **b** Variant classification of *TP53* mutations in PAAD. **c** *TP53* mutations are associated with worse survival prognosis in PAAD. **d** Expression association between *PLK1* and *TP53* in PAAD. PAAD: pancreatic adenocarcinoma

For example, if we are interested in the research of the tumor suppressor gene *TP53* in cancers, we can enter into the “Gene” module to search for the gene. Firstly, we obtain a summary of the *TP53* mean expression levels and somatic mutation rates in 33 cancer types. We find that *TP53* has the highest mutation rate of 91.2% in uterine carcinosarcoma (UCS) and has the second highest mutation rate of 83% in ovarian serous cystadeno-carcinoma (OV). There are ten cancer types that have a *TP53* mutation rate greater than 50% in total (Fig. 1a). Moreover, we can find a summary of the variant classification of *TP53* mutations in cancers, e.g., in pancreatic adenocarcinoma (PAAD), 64 and 12% of *TP53* mutations being missense and frame-shift insertion, respectively (Fig. 1b). Importantly, we can find the associations of *TP53* mutations with survival prognosis in cancers. For example, *TP53* mutations are associated with worse survival (overall and disease free survival) prognosis in PAAD (Fig. 1c). In addition, one could be interested in the expression associations of other genes with *TP53* in cancers, e.g., the expression association between *PLK1* and *TP53* in PAAD (Fig. 1d). In fact, previous studies have shown that *PLK1* interacted with *TP53*, and that p53 dysfunction caused enhanced expression of *PLK1* in cancers [6–9].

#### Functions of the “MicroRNA” module

In the “MicroRNA” module, a user can submit the querying of miRNAs using the human miRNA symbol. TCOA will output the miRNA expression-related data in 33 cancer types. These data include: miRNA expression levels in cancers; expression correlations with genes in cancers; expression correlations with other miRNAs in cancers; differential miRNA expression comparisons between cancer and normal samples (if the miRNA expression data in normal samples are available in TCGA); differential miRNA expression comparisons between different cancer phenotypes such as stage and grade; associations of miRNA expression with survival prognosis in cancers.

**Fig. 2:**
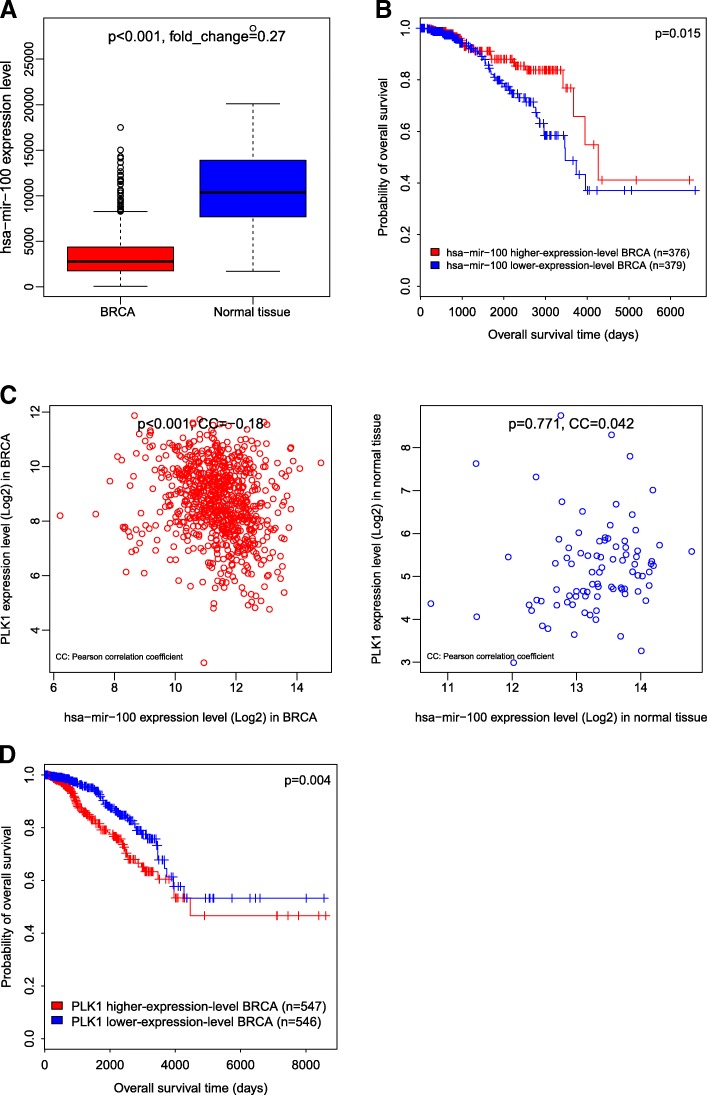
Investigation of hsa-mir-100 in the “MicroRNA” module. **a** hsa-mir-100 has significantly lower expression levels in BRCA than in normal tissue. **b** Elevated expression of hsa-mir-100 is associated with better OS prognosis in BRCA. **c** *PLK1* and hsa-mir-100 have significantly negative expression correlation in BRCA. **d** Elevated expression of *PLK1* is associated with better OS prognosis in BRCA. BRCA: breast invasive carcinoma. OS: overall survival

For example, to explore the human miRNA hsa-mir-100 in cancers, we can submit the querying of hsa-mir-100 in the “MicroRNA” module. Firstly, we obtain a summary of hsa-mir-100 mean expression levels in 33 cancer types. Further, we desire to explore the expression levels of hsa-mir-100 in breast invasive carcinoma (BRCA). In selecting the cancer type, we find that hsa-mir-100 has significantly lower expression levels in BRCA than in normal tissue (Fig. 2a). Moreover, we find that elevated expression of hsa-mir-100 is associated with better overall survival (OS) prognosis in BRCA (Fig. 2b). Furthermore, we explore the expression correlation between gene *PLK1* and hsa-mir-100, and find that *PLK1* and hsa-mir-100 have significantly negative expression correlation in BRCA while have no significant expression correlation in normal tissue (Fig. 2c). This is consistent with previous studies showing that miR-100 could induce apoptosis and cell cycle arrest in cancer by targeting a number of genes including *PIK1* [10, 11]. Accordingly, the TCOA search result shows that elevated expression of *PLK1* is associated with worse OS prognosis in BRCA (Fig. 2d).

#### Functions of the “Cancer” module

In the “Cancer” module, when a user clicks a cancer type, TCOA will output top 50 most frequently mutated genes in the cancer, up-regulated and down-regulated genes, and up-regulated and down-regulated miRNAs in the cancer relative to normal controls. This module also outputs important pathways associated with the highly-expressed genes in the cancer type. TCOA outputs the up-regulated and down-regulated genes or miRNAs depending on the threshold input by users. The threshold includes: fold change of expression levels in cancer compared to normal tissue, and adjusted *p*-value. The adjusted *p*-values (FDR q-values) are calculated by the Benjamini and Hochberg (BH) method [12].

**Fig. 3:**
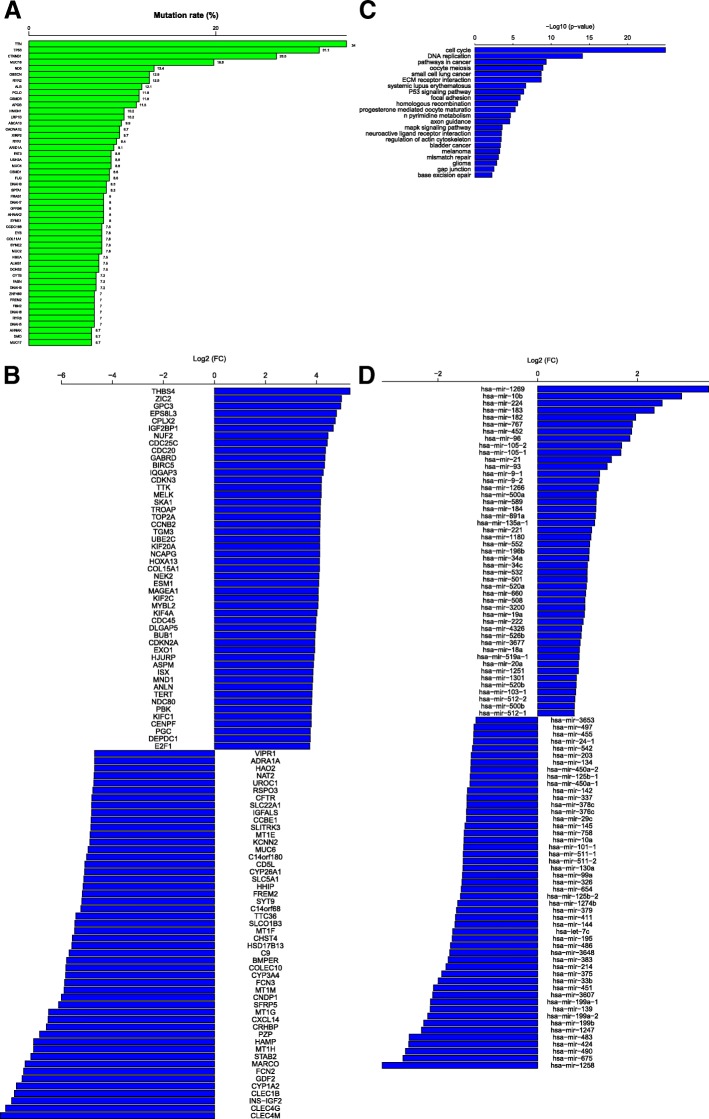
Investigation of LIHC in the “Cancer” module. **a** Mutation rates of the 50 most frequently mutated genes in LIHC. **b** Top 50 up-regulated and top 50 down-regulated genes in LIHC. FC: fold change. FC = gene mean expression levels in cancer / gene mean expression levels in normal tissue. **c** Important pathways associated with the highly-expressed genes in LIHC. **d** Top 50 up-regulated and top 50 down-regulated miRNAs in LIHC. FC = miRNA mean expression levels in cancer / miRNA mean expression levels in normal tissue. LIHC: liver hepatocellular carcinoma. miRNAs: microRNAs

For example, if we submit the querying of liver hepatocellular carcinoma (LIHC) in the module, we will find that TTN has the highest mutation rate of 34%, and *TP53* has the second highest mutation rate of 31.1% in LIHC. The other frequently-mutated genes in LIHC include *CTNNB1*, *MUC16*, *ND5*, *OBSCN*, *RYR2*, *ALB* etc. (Fig. 3a). TCOA shows that *THBS4* has the highest mean expression increase (nearly 40-fold) in LIHC relative to normal tissue. This gene has been shown to be overexpressed in multiple cancer types [13, 14]. The other overexpressed genes in LIHC include *ZIC2*, *GPC3*, *EPS8L3*, *CPLX2*, *IGF2BP1*, *NUF2*, *CDC25C*, *CDC20,* and *GABRD* (Fig. 3b). In contrast, the most down-regulated gene in LIHC is *CLEC4M* which has nearly 335-fold expression decrease compared to normal tissue. This gene encodes a protein that is involved in the innate immune system and is expressed in the endothelial cells of the lymph nodes and liver. Previous studies have shown that *CLEC4M* and its product were down-regulated in LIHC and other cancer types [15, 16]. The other repressed genes in LIHC include *CLEC4G*, *INS-IGF2*, *CLEC1B*, *CYP1A2*, *GDF2*, *FCN2*, *MARCO*, *STAB2*, *HAMP,* and *MT1H* (Fig. 3b). The gene set enrichment analysis of the highly-expressed genes in LIHC shows that the pathways of cell cycle, DNA replication, ECM-receptor interaction, p53 signaling, MAPK signaling, axon guidance, focal adhesion, metabolism, and mismatch repair are enriched in LIHC (Fig. 3c). In addition, TCOA shows that mir-1269, mir-10b, mir-224, and mir-183 are overexpressed in LIHC with more than 4-fold expression increase compared to normal tissue, while mir-1258, mir-675, mir-490, mir-424, mir-483, mir-1247, mir-199b, mir-199a-2, mir-139, mir-199a-1, mir-3607, and mir-451 are underexpressed in LIHC with more than 4-fold expression decrease compared to normal tissue (Fig. 3d).

#### Functions of the “pan-cancer” module

**Fig. 4:**
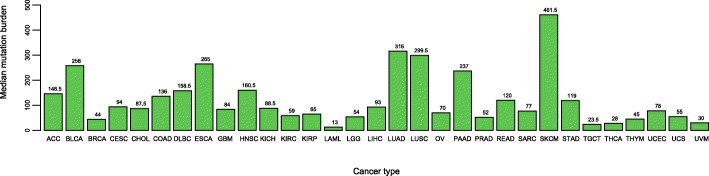
Comparison of tumor mutation burden among different cancer types as shown in the “Pan-cancer” module. BLCA: bladder urothelial carcinoma. BRCA: breast invasive carcinoma. CHOL: cholangiocarcinoma. COAD: colon adenocarcinoma. ESCA: esophageal carcinoma. GBM: glioblastoma multiforme. HNSC: head and neck squamous cell carcinoma. KICH: kidney chromophobe. KIRC: kidney renal clear cell carcinoma. KIRP: kidney renal papillary cell carcinoma. LIHC: liver hepatocellular carcinoma. LUAD: lung adenocarcinoma. LUSC: lung squamous cell carcinoma. PRAD: prostate adenocarcinoma. READ: rectum adenocarcinoma. STAD: stomach adenocarcinoma. THCA: thyroid carcinoma. UCEC: uterine corpus endometrial carcinoma. ACC: adrenocortical carcinoma. CESC: cervical squamous-cell carcinoma and endocervical adenocarcinoma. DLBC: lymphoid neoplasm diffuse large B-cell lymphoma. LAML: acute myeloid leukemia. LGG: brain lower grade glioma. OV: ovarian serous cystadenocarcinoma. PAAD: pancreatic adenocarcinoma. SARC: sarcoma. SKCM: cutaneous melanoma. TGCT: testicular germ cell tumors. UCS: uterine carcinosarcoma. UVM: uveal melanoma. THYM: thymoma

In the “Pan-cancer” module, TCOA outputs the genes consistently up-regulated or down-regulated, pathways significantly up-regulated, and genes whose deregulation is significantly associated with survival prognosis across various cancer types. This module also outputs the genes that are differentially expressed between cancer and normal samples, and between low-advanced and highly-advanced cancers across various cancer types. We refer to early-stage (Stage I-II) or low-grade (Grade I-II) cancers as lowly-advanced cancers, and late-stage (Stage III-IV) or high-grade (Grade III-IV) cancers highly-advanced cancers. A comparison of tumor mutation burden (TMB, defined as the total number of substitutions, regardless of variant type) among different cancer types is also shown in this module (Fig. 4). Figure 4 shows that cutaneous melanoma (SKCM) has the highest median TMB, followed by lung adenocarcinoma (LUAD) and lung squamous cell carcinoma (LUSC). It confirms that TMB was associated with clinical response to immunotherapy [17–19] in that several cancer types with high TMB have shown positive response to immune checkpoint blockade treatment such as melanoma [20] and non-small cell lung cancer (NSCLC) [21]. The results presented in the “Pan-cancer” are mainly based on a recent study by our group [22].

#### Functions of the “Immuno-oncology” module

**Fig. 5:**
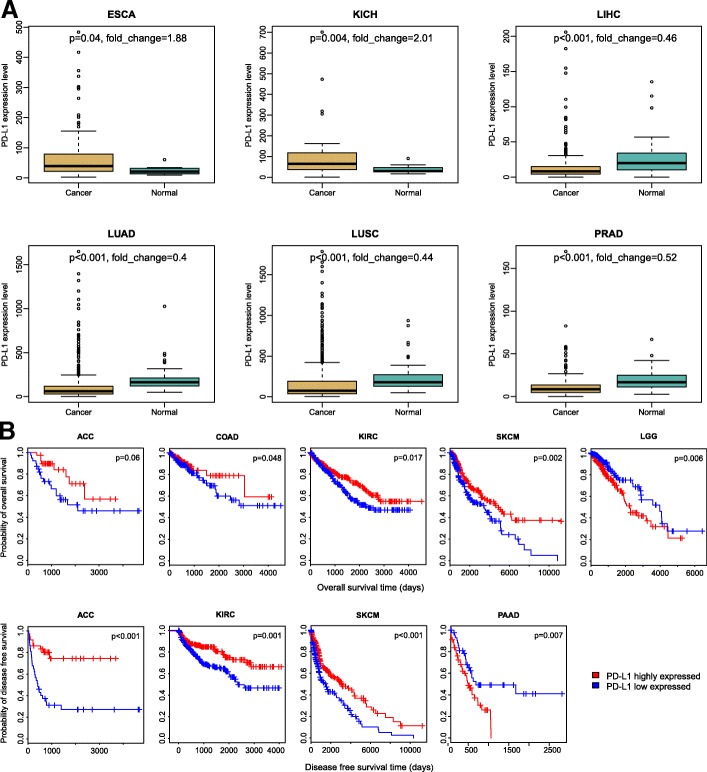
Investigation of *PD-L1* in the “Immuno-oncology” module. **a** Comparison of *PD-L1* gene expression between cancer and normal samples. **b** Associations of *PD-L1* gene expression with survival prognosis in cancers. *PD-L1*: programmed death-ligand 1. ESCA: esophageal carcinoma. KICH: kidney chromophobe. LIHC: liver hepatocellular carcinoma. LUAD: lung adenocarcinoma. LUSC: lung squamous cell carcinoma. PRAD: prostate adenocarcinoma. ACC: adrenocortical carcinoma. COAD: colon adenocarcinoma. KIRC: kidney renal clear cell carcinoma. SKCM: cutaneous melanoma. LGG: brain lower grade glioma. PAAD: pancreatic adenocarcinoma

In the “Immuno-Oncology” module, TCOA provides the querying of 2877 immune-related genes about their expression, mutations and associations with survival prognosis in various cancer types. When a user selects a gene, TCOA will enter into the gene information interface that is the same as that by the “Gene” module querying of that gene. For example, many users could be interested in the gene *PD-L1* whose product plays an important role in cancer immune evasion and is an important target for cancer immunotherapy [23]. TCOA shows that *PD-L1* has significantly higher expression levels in esophageal carcinoma (ESCA) and kidney chromophobe (KICH), while has significantly lower expression levels in LIHC, LUAD, LUSC and prostate adenocarcinoma (PRAD) compared to their normal tissue (Fig. 5a). Interestingly, elevated expression of *PD-L1* is associated with better OS and/or disease free survival (DFS) prognosis in adrenocortical carcinoma (ACC), colon adenocarinoma (COAD), kidney renal clear cell carcinoma (KIRC), and SKCM, while worse OS and/or DFS prognosis in brain lower grade glioma (LGG) and PAAD (Fig. 5b).

#### Functions of the “protein” module

**Fig. 6:**
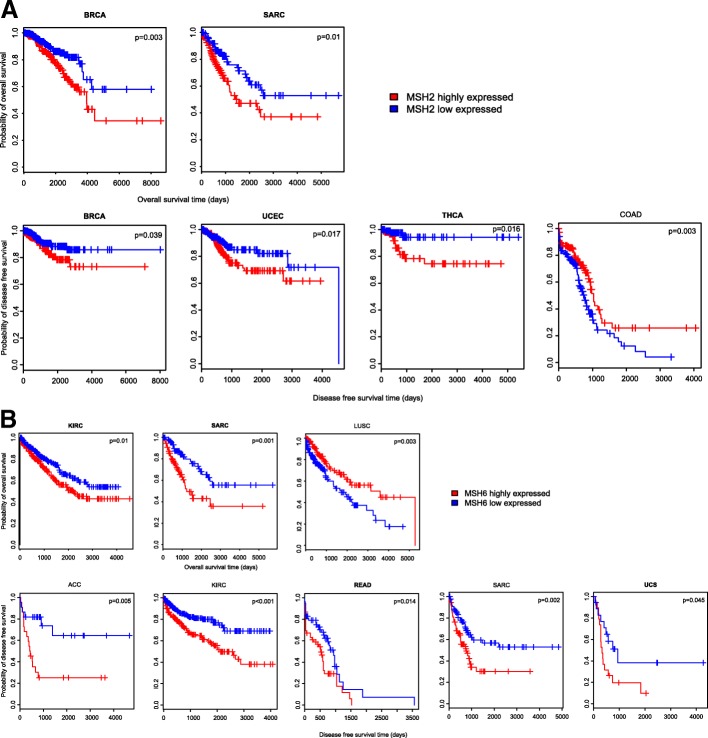
Expression of DNA mismatch repair proteins MSH2 and MSH6 is significantly associated with survival prognosis in various cancer types as shown in the “Protein” module. **a** MSH2 expression is significantly associated with survival prognosis in various cancer types. **b** MSH6 expression is significantly associated with survival prognosis in various cancer types. MSH2: MutS protein homolog 2. MSH6: MutS protein homolog 6. BRCA: breast invasive carcinoma. SARC: sarcoma. THCA: thyroid carcinoma*.* UCEC: uterine corpus endometrial carcinoma*.* COAD: colon adenocarcinoma. ACC: adrenocortical carcinoma. KIRC: kidney renal clear cell carcinoma. READ: rectum adenocarcinoma. UCS: uterine carcinosarcoma. LUSC: lung squamous cell carcinoma

In the “Protein” module, when a user submits the querying of a protein, TCOA will output expression data for the protein in 33 cancer types. These data include: protein expression levels in cancers; protein-gene expression correlation in cancers; differential protein expression comparisons between different cancer phenotypes such as stage and grade; associations of protein expression with survival prognosis in cancers. Figure 6 shows two DNA mismatch repair proteins MSH2 (MutS protein homolog 2) and MSH6 (MutS protein homolog 6) whose expression is significantly associated with survival prognosis in a wide type of cancers. Elevated expression of MSH2 and MSH6 is associated with worse OS and/or DFS prognosis in BRCA, sarcoma (SARC), uterine corpus endometrial carcinoma (UCEC), thyroid carcinoma (THCA), rectum adenocarcinoma (READ), KIRC, UCS and ACC, while is associated with better OS and/or DFS prognosis in LUSC and COAD.

### Computational and statistical analyses

#### Class comparison to identify differentially-expressed genes, miRNAs or proteins

We normalized the TCGA gene and miRNA expression values by log_2_(x + 1) transformation, and used the original downloaded protein expression data since they had been normalized. We compared expression levels of a single gene, miRNA or protein between two classes of samples using Student’s *t* test.

#### Comparison of gene mutation rates among different clinical phenotypes

We compared the gene somatic mutation rates among different clinical phenotypes of cancer patients using Fisher’s Exact Test. Each phenotype was divided into two classes: tumor stage (early stage (Stage I-II) vs. late stage (Stage III-IV)); tumor size (T) (small size (T1–2) vs. large size (T3–4)); lymph nodes (N) (without regional lymph nodes (N0) vs. with lymph nodes (N1–3)); metastasis (M) (no metastasis (M0) vs. metastasis (M1)); grade (low grade (Grade I-II) vs. high grade (Grade III-IV)).

#### Correlation analysis, pathway analysis and survival analysis

We calculated expression correlations of gene-gene, gene-miRNA, miRNA-miRNA and gene-protein by Pearson product-moment or Spearman correlation analysis. We performed pathway analysis of gene sets using the Gene Set Enrichment Analysis (GSEA) software [24]. The KEGG pathways significantly associated with gene sets were displayed (FDR q-value< 0.05). We performed survival analysis of TCGA patients based on gene somatic mutation data, and expression data for genes, miRNAs and proteins, respectively. Kaplan-Meier survival curves were used to show the survival (OS or DFS) differences between gene-mutated cancer patients and gene-wildtype cancer patients, and between gene, miRNA or protein higher-expression-level patients and lower-expression-level patients. Gene, miRNA or protein higher-expression-level and lower-expression-level patients were determined by the median values of expression. If the expression level in a patient was higher than the median value, the patient was classified into the higher-expression-level group; otherwise into the lower-expression-level group. We used the log-rank test to calculate the significance of survival-time differences between two classes of patients.

## Utility and discussion

The TCGA data are providing an invaluable resource for cancer researchers and oncologists. However, a lack of bioinformatics expertise often hinders experimental cancer biologists and oncologists from exploring the TCGA resource. Although a number of tools have been developed for helping cancer biologists and oncologists utilize the TCGA data, these existing tools cannot fully satisfy the large community of experimental cancer biologists and oncologists without bioinformatics expertise. To this end, we developed TCOA with additional functions complementary to these existing tools. TCOA provides fast and straightforward querying of TCGA gene expression, somatic mutations, miRNA expression, protein expression based on a single molecule or cancer type. TCOA provides the querying of expression correlation not only between gene pairs, but also between miRNA pairs, gene and miRNA, and gene and protein. TCOA also provides the querying of the associations of gene, miRNA, or protein expression with survival prognosis in cancers. Moreover, TCOA presents transcriptional profiles across various human cancer types based on the pan-cancer analysis [22]. In addition, TCOA provides the querying of molecular profiles for 2877 immune-related genes in human cancers. These immune-related genes include those that are established or promising targets for cancer immunotherapy such as *CTLA4*, *PD1*, *PD-L1*, *PD-L2*, *IDO1*, *LAG3,* and *TIGIT*. It would be of great interest for cancer researchers and oncologists to query expression, mutations and correlations with cancer survival prognosis of these immune-related genes across various human cancer types.

TCOA will be continuously updated with more functions and modules such as DNA methylation and DNA copy number alteration modules. In addition, for a specific cancer type, one could be interested in molecular alterations across different subtypes. For the immune-related genes, one could be more interested in gene-sets that represent the activities of specific immune cells, functions or pathways [25]. TCOA is expected to provide such functions in future updates.

## Conclusions

TCOA is a useful tool that supplies a number of unique and new functions complementary to the existing tools to facilitate exploration of the TCGA resource.

## Abbreviations

ACC: Adrenocortical carcinoma
BLCA: Bladder urothelial carcinoma
BRCA: Breast invasive carcinoma
CESC: Cervical squamous-cell carcinoma and endocervical adenocarcinoma
CHOL: Cholangiocarcinoma
COAD: Colon adenocarcinoma
DFS: Disease free survival
DLBC: Lymphoid neoplasm diffuse large B-cell lymphoma
ESCA: Esophageal carcinoma
GBM: Glioblastoma multiforme
GSEA: Gene set enrichment analysis
HNSC: Head and neck squamous cell carcinoma
KICH: Kidney chromophobe
KIRC: Kidney renal clear cell carcinoma
KIRP: Kidney renal papillary cell carcinoma
LAML: Acute myeloid leukemia
LGG: Brain lower grade glioma
LIHC: Liver hepatocellular carcinoma
LUAD: Lung adenocarcinoma
LUSC: Lung squamous cell carcinoma
miRNA: MicroRNA
OS: Overall survival
OV: Ovarian serous cystadenocarcinoma
PAAD: Pancreatic adenocarcinoma
PRAD: Prostate adenocarcinoma
READ: Rectum adenocarcinoma
SARC: Sarcoma
SKCM: Cutaneous melanoma
STAD: Stomach adenocarcinoma
TCGA: The Cancer Genome Atlas
TCOA: The Cancer Omics Atlas
TGCT: Testicular germ cell tumors
THCA: Thyroid carcinoma
THYM: Thymoma
UCEC: Uterine corpus endometrial carcinoma
UCS: Uterine carcinosarcoma
UVM: Uveal melanoma

## References

[1] Cerami E (2012). The cBio cancer genomics portal: an open platform for exploring multidimensional cancer genomics data. Cancer Discov.

[2] Gao J (2013). Integrative analysis of complex cancer genomics and clinical profiles using the cBioPortal. Sci Signal.

[3] Koch A (2015). MEXPRESS: visualizing expression, DNA methylation and clinical TCGA data. BMC Genomics.

[4] Tang Z (2017). GEPIA: a web server for cancer and normal gene expression profiling and interactive analyses. Nucleic Acids Res.

[5] Li J (2013). TCPA: a resource for cancer functional proteomics data. Nat Methods.

[6] Wang X, Simon R (2013). Identification of potential synthetic lethal genes to p53 using a computational biology approach. BMC Med Genet.

[7] McKenzie L (2010). p53-dependent repression of polo-like kinase-1 (PLK1). Cell Cycle.

[8] Liu Z, Sun Q, Wang X (2017). PLK1, a potential target for Cancer therapy. Transl Oncol.

[9] Ando K (2004). Polo-like kinase 1 (PLK1) inhibits p53 function by physical interaction and phosphorylation. J Biol Chem.

[10] Li C (2015). Multiple roles of MicroRNA-100 in human Cancer and its therapeutic potential. Cell Physiol Biochem.

[11] Petrelli A (2015). By promoting cell differentiation, miR-100 sensitizes basal-like breast cancer stem cells to hormonal therapy. Oncotarget.

[12] Benjami Y, Hochberg Y. Controlling the false discovery rate: a practical and powerful approach to multiple testing. J R Stat Soc B. 1995;57:289–300.

[13] McCart Reed AE (2013). Thrombospondin-4 expression is activated during the stromal response to invasive breast cancer. Virchows Arch.

[14] Lin X (2016). Associations of THBS2 and THBS4 polymorphisms to gastric cancer in a southeast Chinese population. Cancer Genet.

[15] Yin F (2016). Microarray-based identification of genes associated with cancer progression and prognosis in hepatocellular carcinoma. J Exp Clin Cancer Res.

[16] Liu X (2015). Low expression of dendritic cell-specific intercellular adhesion molecule-grabbing nonintegrin-related protein in lung cancer and significant correlations with brain metastasis and natural killer cells. Mol Cell Biochem.

[17] Snyder A (2014). Genetic basis for clinical response to CTLA-4 blockade in melanoma. N Engl J Med.

[18] Rizvi NA (2015). Cancer immunology. Mutational landscape determines sensitivity to PD-1 blockade in non-small cell lung cancer. Science.

[19] Hugo W (2016). Genomic and transcriptomic features of response to anti-PD-1 therapy in metastatic melanoma. Cell.

[20] Larkin J (2015). Combined Nivolumab and Ipilimumab or monotherapy in untreated melanoma. N Engl J Med.

[21] Herbst RS (2016). Pembrolizumab versus docetaxel for previously treated, PD-L1-positive, advanced non-small-cell lung cancer (KEYNOTE-010): a randomised controlled trial. Lancet (London, England).

[22] Li M, Sun Q, Wang X (2017). Transcriptional landscape of human cancers. Oncotarget.

[23] Chen L, Han X (2015). Anti-PD-1/PD-L1 therapy of human cancer: past, present, and future. J Clin Invest.

[24] Subramanian A (2005). Gene set enrichment analysis: a knowledge-based approach for interpreting genome-wide expression profiles. Proc Natl Acad Sci U S A.

[25] Charoentong P (2017). Pan-cancer Immunogenomic analyses reveal genotype-Immunophenotype relationships and predictors of response to checkpoint blockade. Cell Rep.

[26] Breuer K (2013). InnateDB: systems biology of innate immunity and beyond--recent updates and continuing curation. Nucleic Acids Res.

