# Correction to: The Cancer Omics Atlas: an integrative resource for cancer omics annotations

**DOI:** 10.1186/s12920-018-0393-3

**Published:** 2018-08-30

**Authors:** Qingrong Sun, Mengyuan Li, Xiaosheng Wang

**Affiliations:** 1 Department of Basic Medicine, School of Basic Medicine and Clinical Pharmacy, China Pharmaceutical University, Nanjing, 211198 China; 2 Biomedical Informatics Research Lab, School of Basic Medicine and Clinical Pharmacy, China Pharmaceutical University, Nanjing, 211198 China; 3 Cancer Genomics Research Center, School of Basic Medicine and Clinical Pharmacy, China Pharmaceutical University, Nanjing, 211198 China; 4 Big Data Research Institute, China Pharmaceutical University, Nanjing, 211198 China

## Correction

Following publication of the original article [1], the authors reported an error in Fig. 1A.

Originally, the incorrect figure was published as:

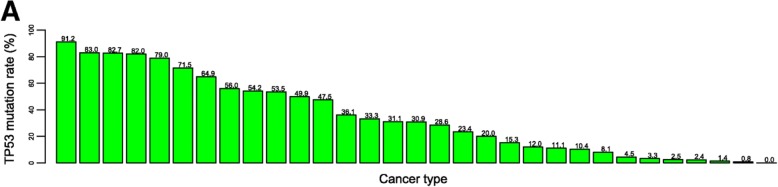



The correct Fig. 1A is:

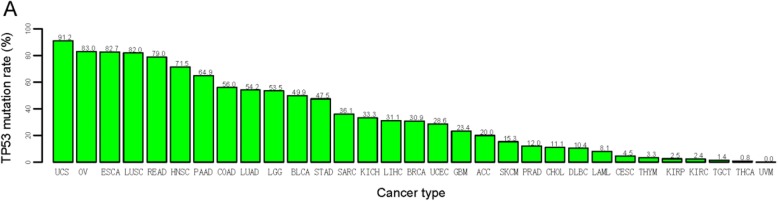



In the reference list, reference 12 contained a spelling error in the first author’s name:

12. Benjami Y, Hochberg Y. Controlling the false discovery rate: a practical and powerful approach to multiple testing. J R Stat Soc B. 1995;57:289–300.

The correct reference is as follows:

12. Benjamini Y, Hochberg Y. Controlling the false discovery rate: a practical and powerful approach to multiple testing. J R Stat Soc B. 1995;57:289–300.
